# Evaluation of Erythroid Disturbance and Thiol-Disulphide Homeostasis in Patients with Psoriasis

**DOI:** 10.1155/2018/9548252

**Published:** 2018-06-05

**Authors:** Suzan Demir Pektas, Gokhan Pektas, Kursad Tosun, Gursoy Dogan, Salim Neselioglu, Ozcan Erel

**Affiliations:** ^1^Department of Dermatology, Mugla Sitki Kocman University Faculty of Medicine, 48000 Mugla, Turkey; ^2^Department of Internal Medicine, Mugla Sitki Kocman University Faculty of Medicine, Training and Research Hospital, 48000 Mugla, Turkey; ^3^Department of Biostatistics, Mugla Sitki Kocman University Faculty of Medicine, 48000 Mugla, Turkey; ^4^Department of Biochemistry, Yildirim Beyazit University, Faculty of Medicine, Ankara, Turkey

## Abstract

This study aims to assess how mean corpuscular volume (MCV), red cell distribution width (RDW), and thiol-disulphide homeostasis are altered in psoriasis patients. This is a cross-sectional review of 76 healthy volunteers and 87 psoriasis patients who were consecutively admitted to the department of dermatology. Psoriasis patients and healthy controls were statistically similar with respect to age, sex, body mass index, blood pressures, and disease duration (*p* > 0.05 for all). When compared to healthy controls, psoriasis patients had significantly higher MCV, RDW, C-reactive protein (CRP), disulphide, disulphide/native thiol, and disulphide/total thiol (*p* < 0.001 for all). However, psoriasis patients had significantly lower native thiol and native thiol/total thiol (*p* = 0.009 and *p* < 0.001, respectively). When compared to healthy controls, the patients with Psoriasis Area Severity Index (PASI) ≤ 10 and patients with PASI > 10 had significantly higher MCV, disulphide, disulphide/native thiol, and disulphide/total thiol (*p* < 0.001 for all). The patients with PASI ≤ 10 and patients with PASI > 10 had significantly lower native thiol/native thiol than healthy controls (*p* < 0.001 for all). The psoriasis patients with PASI > 10 had significantly higher RDW and CRP than healthy controls and patients with PASI ≤ 10 (*p* < 0.001 for all). Disulphide, disulphide/native thiol, disulphide/total thiol, and native thiol/total thiol correlate significantly with both PASI scores and disease duration. Thiol-disulphide homeostasis is enhanced in psoriasis patients. Ongoing inflammation and increased oxidative stress in psoriasis patients also trigger the formation of prooxidants which are neutralized by antioxidants such as thiols. That is why plasma thiol levels are decreased in psoriasis patients.

## 1. Introduction

Psoriasis is a chronic, recurrent, and inflammatory skin disease. It is well known that inflammatory cytokines are increased within the tissues and peripheral circulation of the patients with psoriasis [1, 2]. Neutrophils seem to play a crucial role in the pathogenesis of this disease by contributing to the development and progression of inflammation [3, 4]. Moreover, a few studies reported about the erythroid disturbance in psoriasis patients. These studies showed that red cell distribution width (RDW) increased significantly in psoriasis patients and this significant increase in RDW was attributed to the inflammatory effects of circulating cytokines that modulate erythropoiesis [5, 6].

The pathogenesis of psoriasis is closely associated with oxidative stress [7]. Oxidative stress occurs due to an imbalance between oxidants and antioxidants in favor of the oxidants, leading to a disruption of redox signaling and control and/or molecular abnormalities. Oxidative stress in psoriasis patients is based on lipid peroxidation products such as lipid hydroperoxides, malondialdehyde, and hydroxynonenal, or on enzymatic/nonenzymatic antioxidants [8, 9].

The emergence of oxidative stress depends on the antioxidant defense mechanisms and plasma thiols are strong antioxidants that eliminate free radicals physiologically [3]. The most abundant form of thiol within human cells is glutathione which maintains optimal redox environment for cellular function [10, 11]. Normal levels of reduced glutathione are essential for protecting the cells from the deleterious action of oxidants [7, 12]. Several enzymes such as catalase, superoxide dismutase, and glutathione peroxidase are also involved in the antioxidant defense mechanisms [7].

This study aims to assess the erythroid disturbance and thiol-disulphide homeostasis in patients with psoriasis. This study also aims to investigate whether erythroid disturbance and thiol-disulphide homeostasis correlate with the duration and severity of the disease.

## 2. Materials and Methods

This cross-sectional study was performed in accordance with the guidelines of Helsinki Declaration and it was approved by the local ethical committee. All patients and healthy volunteers gave their written informed consent to participate in the study.

### 2.1. Subjects

This is a prospective review of 76 healthy volunteers and 87 psoriasis patients who were consecutively admitted to the department of dermatology. All psoriasis patients were in the active phase of their disease. The patients who received topical and/or treatment currently and/or within the last three months, the patients with concurrent systemic disorders and other skin diseases, the patients with a habit of smoking and/or alcohol consumption, the patients with any dietary restriction, the patients who used any drugs (including vitamin supplements), pregnant and breastfeeding women were excluded. Disease severity was assessed by Psoriasis Area Severity Index (PASI) and grouped according to PASI score; PASI below or equal to 10 was defined as “mild disease” and above 10 was defined as “moderate-severe disease”. Body mass index (BMI) was calculated by the following formula: body mass index (kg/m^2^) = body weight (kg)/body height^2^ (m^2^)

### 2.2. Laboratory Studies

Venous blood samples were collected into vacuum tubes containing 2 ml K_2_EDTA for complete blood count. It was made sure that none of the samples was icteric or hemolyzed. Hemoglobin, mean corpuscular volume (MCV), RDW, and leukocyte count were measured by an automated blood cell counter (Advia 2120 Hematology Analyzer, Siemens Healthcare Diagnostics Deerfield, IL, USA). Venous blood samples were also collected for serum C-reactive protein (CRP) and thiol-disulphide measurements in the early morning. Then the samples were centrifuged at 3600 rpm for 10 minutes and the obtained sera were kept at −80°C until analysis. Serum CRP levels were measured by turbidimetry (660 nm/700 nm) with a Cobas 6000 Analyzer (Roche Diagnostics, USA). Results were compared with those obtained using typical immunoturbidimetry. Serum thiol-disulphide was measured by using an automatic measurement method (Roche Hitachi Cobas c501 automatic analyzer, Roche Diagnostics, USA) [11]. In order to measure the total amount, disulphide bonds were first reduced to form free functional thiol groups with sodium borohydride and the unused reduced sodium borohydride was treated with formaldehyde in order to prevent the reduction of 5,50- dithiobis-(2 nitrobenzoic) (DTNB). Total thiol groups composed of reduced and native thiol groups were determined after reaction with DTNB. The total thiol content of the sample is measured using modified Ellman reagent. Native thiol content is subtracted from the total thiol content and half of the obtained difference gives the disulphide bond amount. The disulphide parameter can be calculated automatically as half of the difference of the two measured values. Later, the ratios of disulphide/total thiol, disulphide/native thiol and native thiol/total thiol were computed.

### 2.3. Statistical Analysis

Welch *t*-test or Wilcoxon-Mann-Whitney test was applied to the comparison of psoriasis patients with healthy individuals by considering the distribution of the data. Summary statistics of data with normal distribution, i.e., corresponding to the Welch *t*-test, were expressed as mean ± standard deviation. For nonnormal data, it is expressed as median and interquartile range. The ANOVA and Kruskal-Wallis Tests were used to compare the patients with PASI ≤ 10, patients with PASI > 10 and controls. *p* value < 0.05 was considered statistically significant. Bonferroni adjustment was used for multiple comparisons. In comparison with Bonferroni adjustment, the level of significance was considered 0.017. The correlation between parameters, PASI scores, and duration of disease was expressed by Pearson's and Spearman's correlation coefficient. All computational analyzes were performed using the statistical software R [13].

## 3. Results

Table 1 displays the demographic and clinical characteristics of the study groups. The psoriasis patients and healthy controls were statistically similar with respect to age, sex, BMI, and systolic and diastolic blood pressures (*p* > 0.05 for all). The psoriasis patients with PASI ≤ 10 and PASI > 10 had statistically similar disease duration (*p* > 0.05).

Table 2 demonstrates the biochemical characteristics of the healthy controls and psoriasis patients. When compared to healthy controls, psoriasis patients had significantly higher MCV, RDW, CRP, disulphide, disulphide/native thiol, and disulphide/total thiol values (*p* < 0.001 for all). However, psoriasis patients had significantly lower native thiol and native thiol/total thiol than healthy controls (*p* = 0.009 and *p* = 0.001, respectively).

Table 3 compares the biochemical characteristics of the healthy controls, psoriasis patients with PASI ≤ 10 and PASI > 10. When compared with healthy controls, psoriasis patients with PASI ≤ 10 had significantly higher MCV, disulphide, disulphide/native thiol, and disulphide/total thiol (*p* = 0.001 for all). When compared with healthy controls, psoriasis patients with PASI > 10 also had significantly higher MCV, disulphide, disulphide/native thiol, and disulphide/total thiol (*p* = 0.001 for all). The psoriasis patients with PASI ≤ 10 had significantly lower native thiol/native thiol than healthy controls and the patients with PASI > 10 also had significantly lower native thiol/total thiol than healthy controls (*p* = 0.001 for all). The psoriasis patients with PASI > 10 had significantly higher RDW and CRP than healthy controls and psoriasis patients with PASI ≤ 10 (*p* = 0.001 for all).

The MCV, RDW, disulfide, disulfide/native thiol, and disulphide/total thiol correlated positively and significantly with both PASI scores and disease duration (Table 4). On the other hand, native thiol/total thiol correlated negatively and significantly with both PASI scores and disease duration (Table 4). In addition, serum CRP values and leukocyte counts correlated negatively and significantly with disease duration in all psoriasis patients (Table 4).

## 4. Discussion

Chronic inflammation plays a key role in the pathogenesis of psoriasis which is widely viewed as a systemic disease with comorbidities such as metabolic syndrome, obesity, and cardiovascular diseases. Chronic systemic inflammation may also play an important role in the development of related metabolic and vascular disorders. The PASI is a clinical scale which is based on skin findings and which is widely used to evaluate psoriasis patients. However, PASI does not reflect the chronic inflammation [14–16]. That is why, this study evaluates how CRP is altered in patients with psoriasis and whether these markers correlate with the duration and severity of the disease. Moreover, psoriasis patients with any systemic and/or local diseases were excluded to avoid possible confounding factors.

A meta-analysis pointed out that CRP levels were elevated in 24 out of 28 studies which were conducted on patients with psoriasis. However, no relationship between CRP and disease activity could be reported in 15 out of the aforementioned 28 studies [17]. Complying with this meta-analysis, CRP was significantly higher in psoriasis patients than healthy volunteers and in patients with PASI ≥ 10 than patients with PASI < 10 in the present study. Additionally, a negative and significant correlation was found between CRP values and disease duration in psoriasis patients.

Inflammatory cytokines such as E-selectin, intracellular adhesion molecule-1, haptoglobin, interleukins (IL), and tumor necrosis factor-alpha (TNF-*α*) participate in the proliferation of keratinocytes within psoriatic lesions [1, 2, 18]. Interleukin 6 (IL-6) and interferon alpha (IFN-*α*) which cause keratinocyte proliferation and T-cell chemoattraction also induce changes in erythroid progenitor cells, erythropoietin and erythrocyte lifespan [6, 19, 20]. Moreover, it has been reported that erythrocyte damage, aging, and clearance are accelerated in psoriasis patients [7, 21]. Therefore, both enhanced inflammation and erythrocyte damage can lead to increased RDW values in psoriasis patients [6, 21, 22]. As MCV may signify greater differences in erythrocyte size, an increase in MCV can be expected in psoriasis patients who are already exposed to erythrocyte damage in relation to inflammation [22].

Routine complete blood count includes both RDW and MCV which could be used to have an opinion of the general inflammatory burden and erythroid disturbance in psoriasis patients. The increase in these parameters indirectly indicates an exacerbation of the underlying chronic inflammation [23]. Complying with literature, psoriasis patients had significantly higher RDW and MCV values than healthy controls.

The PASI scores correlated positively with RDW and MCV and disease duration correlated positively with MCV in all psoriasis patients and RDW in patients with moderate to severe psoriasis. These findings might be interpreted as an indirect reflection of the flare up in the underlying chronic inflammation. On the other hand, disease duration correlated negatively with serum CRP levels and leukocyte counts in all psoriasis patients. Moreover, PASI scores and disease duration correlated negatively with leukocyte count in patients with moderate to severe psoriasis. These contradictory findings might be due to the previous and long-term administration of immunosuppressive treatment in psoriasis patients.

Psoriasis induces the production of inflammatory cytokines which, in turn, trigger oxidative stress [24–26]. Plasma thiols contain sulphydryl (-SH) groups which are strong antioxidants that physiologically eliminate free radicals. The antioxidant effect of thiol-disulphide homeostasis has a critical role in signal transduction, enzymatic reactions, transcription, detoxification, and apoptosis [3, 11]. Therefore, this study has been designed to specify how thiol-disulphide homeostasis is changed in patients with psoriasis.

Previous studies investigating thiol levels in psoriasis reported discrepant results. Although some studies claimed that plasma thiol levels were decreased in psoriasis patients, plasma thiol levels were found to be unchanged in other studies and plasma thiol levels were found to be increased in the remaining studies [3, 8, 27–30]. A previous study was unable to detect any significant differences in thiol/protein ratios of corneocytes isolated from healthy controls, healthy skin of psoriasis patients, and psoriatic scales [29]. However, Magnus found that thiol levels of psoriatic scales were significantly higher than those of healthy skin tissues [30]. As for the present study, psoriasis patients had significantly higher disulphide, disulphide/native thiol, and disulphide/total thiol but significantly lower native thiol and native thiol/total thiol than healthy controls. The PASI scores correlated positively with disulphide, disulphide/native thiol, and disulphide/total thiol while PASI scores correlated negatively with native thiol/total thiol in psoriasis patients. Although disease duration correlated positively with disulphide, disulphide/native thiol, and disulphide/total thiol values, disease duration correlated negatively with native thiol/total thiol ratios in psoriasis patients. There is a clear disagreement in the results of studies investigating the plasma thiol levels of psoriasis patients. This disagreement may be attributed to the differences in demographic and clinical characteristics of the reviewed patients, variations in biochemical measurement methods, and heterogeneity in study populations.

Disulphide bridges are important in maintaining the physical and chemical stability of mature keratin. Since keratinization is triggered in psoriasis patients, plasma disulphide levels are elevated. As a result of enhanced thiol-disulphide homeostasis, plasma thiol levels are decreased in psoriasis patients. Moreover, ongoing inflammation and enhanced oxidative stress in psoriasis patients continuously cause the formation of prooxidants which are neutralized by antioxidants. Therefore, the consumption of antioxidants may contribute to the reduction in plasma thiol levels. However, these findings should be interpreted carefully as their power is limited by the relatively small cohort size and lack of prospective data. Further research is warranted to clarify how thiol-disulphide homeostasis and inflammatory markers are altered in patients with psoriasis.

## Figures and Tables

**Table 1:** Demographic and clinical characteristics of the study groups.

	Healthy controls (*n* = 76)	Psoriasis patients (*n* = 87)	Patients with PASI ≤ 10 (*n* = 56)	Patients with PASI > 10 (*n* = 31)
Age (years)	32.7 ± 9.7	34.5 ± 10.4	34.4 ± 9.9	34.8 ± 11.4
Sex (Male/Female)	42/34 (55.3%/44.7%)	53/34 (60.9%/39.1%)	37/19 (66.1%/33.9%)	16/15 (51.6%/48.4%)
Systolic blood pressure (mmHg)	119.1 ± 12.8	117.5 ± 11.7	120.6 ± 14.3	115.7 ± 9.8
Diastolic blood pressure (mmHg)	74.5 ± 7.3	76.6 ± 7.4	80.6 ± 7.7	74.4 ± 6.4
Body mass index (kg/m^2^)	23.6 ± 0.8	23.9 ± 0.7	23.9 ± 0.7	23.9 ± 0.8
Disease duration (months)		28.5 ± 8.9	28.5 ± 9.1	28.5 ± 8.6
Psoriasis Area Severity Index (PASI)		10.2 ± 3.7	8.1 ± 1.4	14.1 ± 3.6

**Table 2:** Hematologic, inflammatory, and disulphide-homeostasis studies of the healthy controls and psoriasis patients.

		Healthy controls (*n* = 76)	Psoriasis patients (*n* = 87)	*p*-value
Hemoglobin (g/dL)	Median; IQR	13.5; 1.1	13.2; 1.6	0.160
	(min, max)	(12.1, 15.6)	(12.0, 15.9)	
Mean corpuscular volume (fL)	Mean ± SD	84.76 ± 3.87	88.45 ± 3.45	0.001_ _^*∗*^
	(min, max)	(79, 93)	(80, 95)	
Red cell distribution width (%)	Median; IQR	12.8; 0.8	13.2; 1.1	0.001_ _^*∗*^
	(min, max)	(11.8, 14.6)	(12.0, 16.1)	
Leukocyte count (×10^9^/L)	Mean ± SD	7.4 ± 1.3	7.3 ± 2.0	0.791
	(min, max)	(3.9, 9.8)	(1.2, 12.0)	
C-reactive protein (mg/dl)	Median; IQR	3.5; 1.7	9.0; 6.5	0.001_ _^*∗*^
	(min, max)	(2.2, 18.9)	(2.5, 22.0)	
Native thiol (*µ*mol/L)	Median; IQR	423.05; 89.5	384.36; 118.1	0.009_ _^*∗*^
	(min, max)	(129.7, 491.9)	(174.7, 550.9)	
Total thiol (*µ*mol/L)	Median; IQR	456.05; 84.2	425.17; 115.1	0.074
	(min, max)	(139.8, 533.2)	(228.1, 610.5)	
Disulphide (*µ*mol/L)	Median; IQR	15.03; 8.80	18.75; 6.79	0.001_ _^*∗*^
	(min, max)	(1.9, 36.0)	(6.5, 36.0)	
Disulphide/native thiol (%)	Median; IQR	3.59; 1.90	5.09; 1.90	0.001_ _^*∗*^
	(min, max)	(0.6, 12.5)	(2.3, 15.3)	
Disulphide/total thiol (%)	Median; IQR	3.35; 1.64	4.62; 1.56	0.001_ _^*∗*^
	(min, max)	(0.6, 10.0)	(2.2, 11.7)	
Native thiol/total thiol (%)	Median; IQR	93.29; 3.28	90.76; 3.12	0.001_ _^*∗*^
	(min, max)	(80.0, 98.7)	(76.6, 95.6)	

IQR: interquartile range, SD: standard deviation; ^*∗*^*p* < 0.05 was accepted to be statistically significant.

**Table 3:** Hematologic, inflammatory, and disulphide-homeostasis studies of the groups.

		Healthy controls (*n* = 76)	Patients with PASI ≤ 10 (*n* = 56)	Patients with PASI > 10 (*n* = 31)	*p*-value
Hemoglobin (g/dL)	Median; IQR	13.5; 1.1	13.10; 0.9	13.50; 2.3	0.133
	(min, max)	(12.1, 15.6)	(12.1, 15.8)	(12.0, 15.9)	
Mean corpuscular volume (fL)	Mean ± SD	84.76 ± 3.87	89.0 ± 4.3	89.0 ± 5.0	0.001_ _^†‡^
	(min, max)	(79, 93)	(80, 95)	(83, 95)	
Red cell distribution width (%)	Median; IQR	12.8; 0.8	13.0; 0.7	14.5; 1.2	0.001_ _^§‡^
	(min, max)	(11.8, 14.6)	(12.0, 14.2)	(13.4, 16.1)	
Leukocyte count (×10^9^/L)	Mean ± SD	7.4 ± 1.3	7.4 ± 2.2	7.0 ± 1.8	0.495
	(min, max)	(3.9, 9.8)	(1.4, 12.0)	(1.2, 11.3)	
C-reactive protein (mg/dl)	Median; IQR	3.5; 1.7	8.9; 4.4	12.6; 6.35	0.001_ _^§‡^
	(min, max)	(2.2, 18.9)	(2.5, 21.0)	(3.1, 22.0)	
Native thiol (*µ*mol/L)	Median; IQR	423.05; 89.5	385.83; 121.72	379.40; 94.05	0.056
	(min, max)	(129.7, 491.9)	(174.7, 550.9)	(216.7, 538.8)	
Total thiol (*µ*mol/L)	Median; IQR	456.05; 84.2	423.60; 119.36	433.00; 94.30	0.199
	(min, max)	(139.8, 533.2)	(228.1, 610.5)	(249.6, 569.2)	
Disulphide (*µ*mol/L)	Median; IQR	15.03; 8.80	19.77; 5.79	19.95; 5.33	0.001_ _^†‡^
	(min, max)	(1.9, 36.0)	(6.5, 36.0)	(11.1, 30.1)	
Disulphide/native thiol (%)	Median; IQR	3.59; 1.90	5.00; 1.74	5.09; 2.58	0.001_ _^†‡^
	(min, max)	(0.6, 12.5)	(2.3, 15.3)	(2.5, 12.8)	
Disulphide/total thiol (%)	Median; IQR	3.35; 1.64	4.55; 1.43	4.62; 2.09	0.001_ _^†‡^
	(min, max)	(0.6, 10.0)	(2.2, 11.7)	(2.4, 10.2)	
Native thiol/total thiol (%)	Median; IQR	93.29; 3.28	90.90; 2.86	90.76; 4.17	0.001_ _^†‡^
	(min, max)	(80.0, 98.7)	(76.6, 95.6)	(79.6, 95.2)	

PASI: Psoriasis Area Severity Index.  ^†^There was statistical significance between healthy controls and psoriasis patients with PASI ≤ 10 (*p* < 0.001).  ^‡^There was statistical significance between healthy controls and psoriasis patients with PASI > 10 (*p* < 0.001).  ^§^There was statistical significance between psoriasis patients with PASI ≤ 10 and PASI > 10 (*p* < 0.001).

**Table 4:** Correlation of biochemical markers with disease duration and severity in psoriasis patients.

	Total PASI in all patients		Disease duration in all patients		PASI ≤ 10		PASI > 10		Disease duration in patients with PASI ≤ 10		Disease duration in patients with PASI > 10	
	*r*	*p*	*r*	*p*	*r*	*p*	*r*	*p*	*r*	*p*	*r*	*p*
MCV	0.29	0.007	0.63	0.001	0.23	0.091	0.52	0.003	0.64	0.001	0.59	0.001
RDW	0.82	0.001	0.58	0.004	0.32	0.016	0.71	0.001	−0.10	0.442	0.46	0.010
Leukocyte	−0.17	0.115	−0.29	0.007	0.09	0.518	−0.38	0.038	−0.31	0.020	−0.24	0.196
CRP	0.02	0.870	−0.25	0.021	0.02	0.882	0.02	0.933	−0.25	0.059	−0.23	0.207
Disulphide	0.29	0.007	0.85	0.001	0.31	0.020	0.61	0.001	0.83	0.001	0.89	0.001
Disulphide/Native thiol	0.27	0.011	0.70	0.001	0.17	0.197	0.56	0.001	0.70	0.001	0.72	0.001
Disulphide/Total thiol	0.28	0.009	0.72	0.001	0.20	0.130	0.55	0.001	0.71	0.001	0.74	0.001
Native thiol/Total thiol	−0.28	0.009	−0.72	0.001	−0.20	0.130	−0.55	0.001	−0.71	0.001	−0.74	0.001

## Data Availability

The data used to support the findings of this study are available from the corresponding author upon request.
